# Neurobrucellosis: the great mimicker

**DOI:** 10.1590/0037-8682-0567-2021

**Published:** 2022-04-08

**Authors:** Cristiane Nascimento Soares, Abraão Iuri Medeiros Angelim, Carlos Otavio Brandão, Roberto Queiroz Santos, Ravi Mehta, Marcus Tulius Teixeira da Silva

**Affiliations:** 1 Hospital Federal dos Servidores do Estado, Departamento de doenças infecciosas, Rio de Janeiro, RJ, Brasil.; 2 Hospital Federal dos Servidores do Estado, Departamento de Neurologia, Rio de Janeiro, RJ, Brasil.; 3 Laboratório Neurolife, Rio de Janeiro, RJ, Brasil.; 4 Hospital Federal dos Servidores do Estado, Departamento de Radiologia, Rio de Janeiro, RJ, Brasil.; 5 Imperial College London, Department of Infectious Diseases, London, England.; 6 Instituto de Doenças Infecciosas Evandro Chagas, Laboratório de Pesquisa Clínica em Neuroinfecções, Rio de Janeiro, RJ, Brasil.

**Keywords:** Neurobrucellosis, Chronic meningitis, Brucellosis, Neurological manifestations

## Abstract

Neurobrucellosis is caused by bacteria of the genus *Brucella* and is responsible for several clinical manifestations, making diagnosis challenging. The most common route of infection is through the consumption of unpasteurized or raw dairy products such as fresh milk, butter, and cheese. As neurological complications can develop chronically, they are frequently misdiagnosed as other infections, such as tuberculosis. This report reviews the clinical manifestations, diagnostic approach, treatment, and prognosis of neurobrucellosis, illustrating a case of chronic intracranial hypertension and meningoencephalitis secondary to brucellosis. The clinical presentation of brucellosis can mimic several systemic diseases, resulting in diagnostic delays and clinical complications. A high degree of suspicion is required, and neurobrucellosis should always be considered in the differential diagnosis of chronic meningitis.

## INTRODUCTION

Brucellosis is also known as “undulant fever,” “Mediterranean fever,” or “Malta fever,” and is a zoonotic infection caused by the bacteria of genus *Brucella*. It is a gram-negative, intracellular, aerobic bacteria, of which there are six species, with four causing brucellosis in humans (*Brucella abortus*, *B. melitensis*, *B. suis*, and *B. canis*)^1^. Human beings are usually dead-end hosts, and the main animal reservoirs are cattle, sheep, goats, and pigs. Human brucellosis is a multisystem disease involving the liver, spleen, bone marrow, lymph nodes, nervous, musculoskeletal, cardiovascular, gastrointestinal, and genitourinary systems^1^. 

The neurological presentation includes meningitis, meningoencephalitis, encephalitis, cranial neuropathies, intracranial hypertension, sinus thrombosis, radiculitis, peripheral neuropathy, myelitis, and psychiatric manifestations ^2^ ^,^ ^3^. The disease can be insidious and present in many atypical forms, leading to delays in clinical recognition. Neurobrucellosis is most commonly diagnosed 2-12 months after symptom onset of symptoms^4^. As neurological complications can develop chronically, they are frequently misdiagnosed as other infections, such as tuberculosis. In this report, we review the main aspects of neurobrucellosis and describe a case of chronic intracranial hypertension and meningoencephalitis caused by *Brucella*.

## EPIDEMIOLOGY AND PHYSIOPATHOLOGY

The World Health Organization estimates an incidence of 500,000 brucellosis cases/year^5^. It is found globally, but its incidence is most significant mainly in the Mediterranean basin (Portugal, Spain, Southern France, Italy, Greece, Turkey, and North Africa), Arabian Peninsula, Indian subcontinent, and parts of Mexico, Central, and South America^6^. Despite its global distribution, it is often unrecognized and frequently unreported.

The most common way to be infected is by eating or drinking unpasteurized or raw dairy products such as fresh milk, butter, and cheese^7^. *Brucella* can survive in these products for two weeks to three months. Other transmission routes include the ingestion of undercooked meat, contamination via wounds in the skin/mucous membranes through contact with infected animals, and inhalation^4^. Direct person-to-person spread of brucellosis is rare, as is transmission via sexual contact, tissue transplantation, or blood transfusion.

Shortly after infection, *Brucella* causes bacteremia and can spread to different systems in the body. Most of the symptoms and neurological manifestations are evidenced after this period of septicemia, in a more subacute manner, and can be expressed from months to years after infection onset^8^. The acute phase of the disease begins after an incubation period of 2-4 weeks, with fever, pain, and diaphoresis. Fever is usually vespertine and intermittent. Brucellosis can cause chronic infection if it persists for more than two months. The most common system involved is the osteoarticular system, followed by the nervous system. Additionally, hematologic, cardiopulmonary, and genitourinary manifestations are found^9^. 

Although the mechanisms of neuropathophysiology in neurobrucellosis remain unclear, three hypotheses exist a) a direct neuropathic effect, b) deleterious cytokine or endotoxin release, and c) an inflammatory/immunologic host reaction to *Brucella* within the nervous system^10^. As brucellosis is usually a chronic infection, nervous system invasion can occur secondary to the persistence of intracellular microorganisms. Preexisting host immunosuppression is a significant risk factor, but the disease can also occur in healthy individuals. The development of neurobrucellosis is also associated with age and prolonged time of infection^11^.

## NEUROLOGICAL MANIFESTATIONS

Neurobrucellosis represents a huge challenge in infectious diseases owing to its range of clinical manifestations^12^. Neurological complications can be classified mainly by the involvement of the central or peripheral nervous systems^12^ ^,^ ^13^.

The most common form is involvement of the central nervous system, with meningitis being the predominant manifestation. It can occur in both acute and chronic conditions. In a review of 187 cases included in 37 publications, meningeal irritation was the most common symptom, reported in 37% of the cases^12^. Meningovascular complications such as mycotic aneurysms, ischemic strokes, and subarachnoid hemorrhages are also frequently described.

Additionally, cranial nerve (CN) involvement is common, as well as complications of the arachnoid drainage system, which can cause intracranial hypertension. The vestibulocochlear nerve is the most commonly involved CN, with the clinical development of sensorineural hearing loss, as demonstrated in several case reports^14^. The sixth and seventh CNs were the second and third most affected, respectively. However, involvement of the sixth CN, leading to diplopia in neurobrucellosis, has rarely been described.

Other complications, such as psychiatric symptoms, are rare; the most commonly observed are depression, personality changes, euphoria, and psychosis^15^. An analysis of 82 patients with brucellosis showed that 18 patients (21%) with neurological manifestations had cognitive disorders. After appropriate treatment, these patients demonstrated improvements in cognitive symptoms^16^ ^,^ ^17^.

Myelitis usually develops due to direct or systemic spinal cord infection or following a systemic infection^18^. Similar to other infections, brucellosis can trigger an aberrant immune response and lead to recurrent transverse myelitis^19^. Although oligoclonal bands in the CSF of patients with neurobrucellosis supports this autoimmune theory, it is not unique to this infection^20^. Commitment of the spinal cord can also occur because of compression of the abscess, granuloma, or spinal root involvement.

Peripheral nervous system involvement occurs in 7% of neurobrucellosis cases^21^. Acute, subacute, or chronic polyradiculoneuropathies, some without sensory involvement or mimicking Guillain-Barré syndrome, have been described in case reports^21^ ^,^ ^22^. 

## DIAGNOSIS

Because neurobrucellosis does not present a typical clinical picture^23^, its diagnosis is puzzling. The most commonly used diagnostic criteria are as follows: (1) symptoms and signs suggestive of neurobrucellosis not explained by other neurological diseases, (2) positive CSF culture for *Brucella* organisms or positive *Brucella* IgG agglutination titer in the blood, (3) presence of lymphocytic pleocytosis and increased protein in CSF, and (4) response to specific antibiotics with a significant improvement in CSF parameters^24^. All four criteria are required for the diagnosis of neurobrucellosis.

CSF analysis is not specific to neurobrucellosis since the same findings are seen in several other causes of chronic meningitis, such as tuberculosis and neurocryptococcosis. These infections usually present with elevated CSF protein levels, low glucose levels, and pleocytosis (predominantly lymphocytes)^25^ ^,^ ^26^.

Although serology is vital for diagnosing neurobrucellosis, in endemic areas such as Turkey, positive serology at a low titer can represent a previous infection^27^. Several serological methods with different sensitivities can be used to investigate whether a patient has been or is exposed to *Brucella* (Table 1)^28^. These include the rose bengal test (RBT), standard tube agglutination (STA), ELISA, complement fixation, indirect Coombs, and immunocapture agglutination (Brucella Capt). Rose Bengal is a simple and fast screening method used mainly in epidemiological studies. It consists of an agglutination test that uses rose-stained Brucella antigen. It is useful for diagnosing acute brucellosis, although there is a high rate of false-negative results in chronic cases. When positive, the results must be confirmed using other tests. Similar to STA, the indirect Coombs test uses visual agglutination using an agglutinoscope or a drop on a slide examined under a microscope. It allows detection of nonagglutinant IgG antibodies, which are associated with slow-developing infection, missing around 7% of cases when compared to ELISA^29^.

**TABLE 1:** Sensitivity of laboratorial methods:

	Serum Sensitvity	CSF Sensitvity
Standard tube Agglutination	94%	78%
Rose Bengal test	96%	71%
ELISA IgM	70%	80%
ELISA IgG	91%	80%
Automated Culture	37%	25%

Ps1: The CSF sensitivity of conventional culture range 9%.

Ps2: Unfortunately, specificities were not available in the literature.

The STA test is considered the cornerstone of brucellosis diagnosis and usually becomes positive during the second to the third week of illness^29^. Although STA remains the most popular and used test for routine diagnostic practice, ELISA has a higher sensitivity and specificity and is the best choice for diagnosing neurobrucellosis and complicated cases. ELISAs have been widely used in chronic cases of brucellosis^30^.

Regarding serological tests, it has been recommended that a combination of two different tests should be performed (for example, STA and Coombs or STA and ELISA) to increase diagnostic accuracy^30^. 

A definite diagnosis of brucellosis relies on isolating bacteria from the blood, CSF, bone marrow, or other tissue cultures. However, the specificity of this test is very low, ranging from 20 to 28% and sensitivity of 37%^4^ ^,^ ^12^ ^,^ ^31^. In a study of 1,028 cases of brucellosis, the CSF culture had the lowest isolation rate (10 %)^32^. Consequently, serological tests should be considered the mainstay of diagnosis of neurobrucellosis. Automated culture, in which bacterial multiplication is detected by monitoring CO2 production, has a higher sensitivity than conventional culture in CSF samples, albeit still low at 25%^32^. 

Biomolecular diagnostic methods (real-time PCR) are useful for both diagnosis and follow-up of neurobrucellosis^29^. Despite their great potential, molecular assays have a variable sensitivity range (50-100%) and specificity (60-98%), requiring further standardization to improve reliability^29^.

The radiological presentations of neurobrucellosis may also differ. It can present as normal, inflammatory (abnormal meningeal enhancement), white matter changes, or vascular changes^33^. White matter changes are non-specific and can easily mimic other inflammatory disorders or infectious diseases^34^. According to the Istanbul-3 study, 45% of neurobrucellosis cases showed changes on MRI. These include meningeal inflammation with post-contrast enhancement, cranial nerve involvement, brain abscesses, spinal nerve root enhancement, arachnoiditis, granulomas, white matter, and vascular changes^34^. The major differential diagnoses in MRI are infectious processes with a skull base predominance, such as tuberculosis, or granulomatous diseases, such as sarcoidosis.

## TREATMENT

*Brucella* can effectively evade the immune response and easily spread throughout the body. Infection can be challenging to cure and often relapse^35^. There is no consensus on the choice of antibiotic, dose, and duration of antimicrobial treatment for neurobrucellosis, and there are no randomized controlled trials. Therefore, adequate antimicrobial CNS penetration is required. A combination of three antibiotics is commonly used until the clinical manifestations vanish and the CSF returns to normal. The duration of antibiotic therapy depended on these factors^3^. Usually, ceftriaxone 4 g/day (due to its excellent CNS penetration) for the first 4-6 weeks, in addition to rifampin and doxycycline for at least 12 weeks, is considered the first-line therapy. A combination of doxycycline, rifampin, and trimethoprim/sulfamethoxazole (TMP-SMX) is another practical regimen that can be used for at least 12 weeks^36^. Patients must be followed-up every three months due to the possibility of relapse. If CSF remains abnormal or clinical manifestations have not subsided, treatment is usually continued for six months^5^. The relapse rate following treatment is approximately 5 to 15%, usually after the end of the first six months of treatment. Therapeutic failures are frequently associated with a short course of antibiotic treatment owing to the persistence of intracellular bacteria^18^.

Corticosteroids appear to protect tissues from the effects of bacterial toxins and reduce the incidence of long-term complications; however, no controlled study has validated the use of steroids in brucellosis. Evidence for the effectiveness of steroid therapy in brain involvement comes from case reports, especially in demyelinating diseases. Steroids have also been used in cases with severe presentations such as arachnoiditis, cranial nerve involvement, myelopathy, intracranial pressure, optic neuritis, or papilledema.

The mortality rate of neurobrucellosis in the post-antibiotic era is 0%-5.5%. One of the most common permanent deficits is deafness (20-30%)^37^. 

## CASE DESCRIPTION

A 21-year-old Brazilian man presented with a headache in February 2019 associated with fatigue, weight loss, fever, and sweats. Three months later, he developed blurred vision in his right eye and left lower-limb paresis. The patient was a salesman with a history of bronchitis. He had no history of travel or contact with animals. 

**FIGURE 1A:**
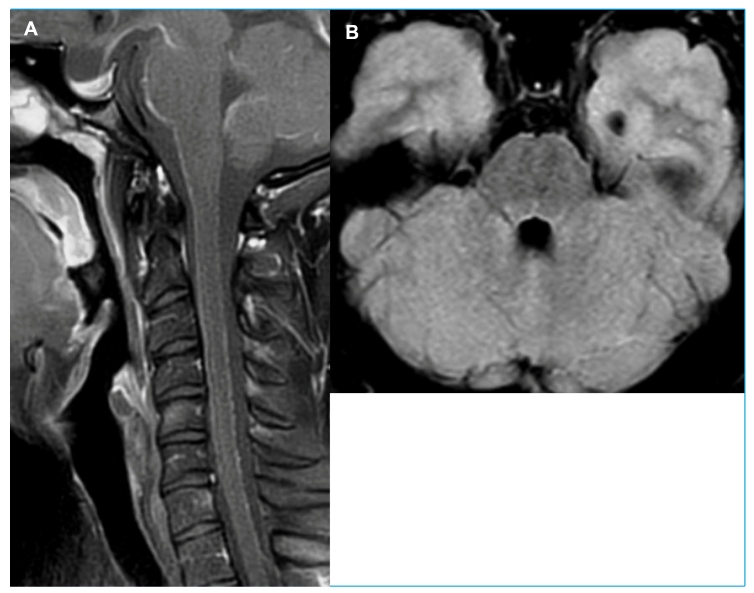
Sagittal T1-weighted fat suppressed shows diffuse leptomeningeal thickening along the surface of the spinal cord and brainstem. **FIGURE 1B:** Axial FLAIR image shows linear hyperintensity along the surface of the pons, indicating leptomeningeal thickening.

He was hemodynamically stable with no signs of meningeal irritation. Examination showed left distal hemiparesis and papilledema in his right eye. Brain MRI showed leptomeningeal enhancement along the pons, medulla, and cervical spinal cord, as well as signs of intracranial hypertension (Figure 1). Cerebrospinal fluid (CSF) was collected by lumbar puncture and showed an opening pressure of 40 cm H2O, 154 white cells/mm^3^ (84% lymphocytes), glucose 21 mg/dl, elevated lactate (28 mg/dl), and total protein of 108 mg/dl. Bacterial and fungal cultures and immunological testing for syphilis (VDRL) were negative, as were those for human T lymphotropic virus I (HTLV-I), herpes simplex virus, varicella-zoster virus, cytomegalovirus, HIV1/2 antibodies, angiotensin-converting enzyme level (ACE), histoplasmosis, and Cryptococcus antigen latex agglutination. PCR results for *M. tuberculosis* were also negative.

Despite negative PCR results, the patient underwent an empirical treatment trial for tuberculous meningitis. Rifampin, isoniazid, pyrazinamide, ethambutol, prednisone, and acetazolamide were prescribed. After two months, his symptoms disappeared. New CSF analysis was performed seven months after the treatment onset. It showed an open pressure of 28 cm H2O, pleocytosis of 79 cells (72% of lymphocytes), the glucose of 24 mg/dl, high lactate level, and 122 mg/dl total protein. After almost one year of treatment, a third CSF sample was collected, but the same abnormal parameters were found. Despite the clinical improvement, we decided to look for another cause of chronic meningitis. Computed tomography findings of the thorax, abdomen, and pelvis were normal. Laboratory tests, including rheumatic and liver screening, IgG4 and ACE levels, and biochemical tests, were all negative. Standard agglutination tests on blood revealed Brucella agglutination titers of 1:80. The blood culture was negative for *Brucella*. 

Therefore, we decided to treat the brucellosis with rifampin (600 mg/day), sulfamethoxazole/trimethoprim (1,600/320 mg/day), and doxycycline (200 mg/day). After three months, his CSF showed considerable improvement, with a decrease in pleocytosis (29 cells) and protein level (85 mg/dl) and normalization of glucose and lactate levels. CSF immunological testing for brucellosis revealed a negative IgM antibody, although a positive IgG antibody was detected (enzyme immunoassay). Brucellosis cultures of the cerebrospinal fluid were negative. The CSF was completely normal after ten months of treatment. Repeat MRI showed resolution of leptomeningeal enhancement. The prednisone and acetazolamide were tapered and discontinued. 

Clinically, the patient remained with a visual deficit in the right eye due to optic nerve atrophy**.** Our patient’s occupation did not put him at high risk of brucellosis, nor did he report contact with high-risk animals. We suspected he was infected with incompletely sterilized milk/cheese or poorly cooked meat.

## CONCLUSION

As the clinical presentation of neurobrucellosis is variable, it is often misdiagnosed. Brucellosis can clinically mimic any systemic disease, resulting in diagnostic delays and increased complications.

Eradication of the disease in humans can only be achieved by controlling the disease in animals, which requires an integrated collaboration between veterinary and human public health. 

## SEARCH STRATEGY AND SELECTION CRITERIA

We searched PubMed and Scopus for articles on neurobrucellosis from database inception from August 2015 to August 2020, using the terms neurobrucellosis and brucellosis in combination with “neurological,” “nervous system,” “meningoencephalitis,” “meningitis,” “encephalitis,” “peripheral neuropathy,” “neuritis,” “epidemiology,” “diagnosis,” “treatment” modified as per requirements for the search tool of each database. Only papers published in English were reviewed. Publications from the authors’ collections were also used. Articles were included based on relevance and originality with regard to the topics covered in this review.
